# Functional Training Mitigates Reduced Circulating Indole‐3‐Lactate Levels in Persons With Relapsing–Remitting Multiple Sclerosis

**DOI:** 10.1111/apha.70166

**Published:** 2026-01-28

**Authors:** Tiffany Wences Chirino, Frederike Adammek, Sergen Belen, Matteo Winker, Sebastian Proschinger, Annette Rademacher, Marit L. Schlagheck, Alexander Schenk, Marie Kupjetz, David Walzik, Clemens Warnke, Marcel Reuter, Friederike Rosenberger, Tim Meyer, Adrian McCann, Per Magne Ueland, Niklas Joisten, Philipp Zimmer

**Affiliations:** ^1^ Sports Medicine Research Group, Institute for Sport and Sport Science, TU Dortmund University Dortmund Germany; ^2^ Department for Molecular and Cellular Sports Medicine Institute of Cardiovascular Research and Sports Medicine, German Sport University Cologne Cologne Germany; ^3^ Asklepios MVZ Bayern GmbH Cham Germany; ^4^ Department of Neurology Faculty of Medicine and University Hospital Cologne Cologne Germany; ^5^ German University of Applied Sciences for Prevention and Health Management Saarbrücken Germany; ^6^ Institute of Sports and Preventive Medicine, University of Saarland Saarbrücken Germany; ^7^ Bevital AS Bergen Norway

**Keywords:** exercise, gut microbiota, indoles, multiple sclerosis, tryptophan

## Abstract

**Aim:**

Indoles are tryptophan (Trp)‐derived metabolites that are produced by the gut microbiota and may influence the gut‐microbiota‐brain axis in multiple sclerosis (MS). Indole‐3‐lactate (ILA) is reduced in persons with MS and improves MS clinical scores in animal models via its anti‐inflammatory remyelinating properties. The ILA/indole‐3‐acetate (IAA) (ILA/AA) index is considered a neuroprotection index. Physical exercise and diet can modify gut microbiota and indole metabolism.

**Methods:**

This secondary analysis of a randomized control trial aimed to assess the effects of acute and chronic exercise on serum indoles in relapsing–remitting MS (RRMS). Thirty‐one RRMS patients (≥ 70% session attendance) completed a 10 week multimodal functional training (60 min, 3×/week) vs. a waitlist control group. Blood samples were collected at baseline and compared to a matched healthy control group, and after 10 weeks for the assessment of chronic effects. Additionally, acute effects of a single bout of exercise were assessed with a blood sample before, during, and immediately after one interim training session. Serum indole concentrations were measured using LC–MS/MS.

**Results:**

Baseline indole levels in RRMS patients differed from those of matched healthy controls, and reduced ILA levels were observed. The 10 week intervention increased the ILA/IAA index, while a single exercise bout induced an increase in both ILA and ILA/IAA.

**Conclusion:**

Multimodal functional training over 10 weeks led to an improved ILA/IAA index suggesting a neuroprotective shift in gut microbiota composition, and a single bout acutely increases the circulating level of ILA. Study registration number: DRKS00017091.

## 1 Introduction

**FIGURE 1:**
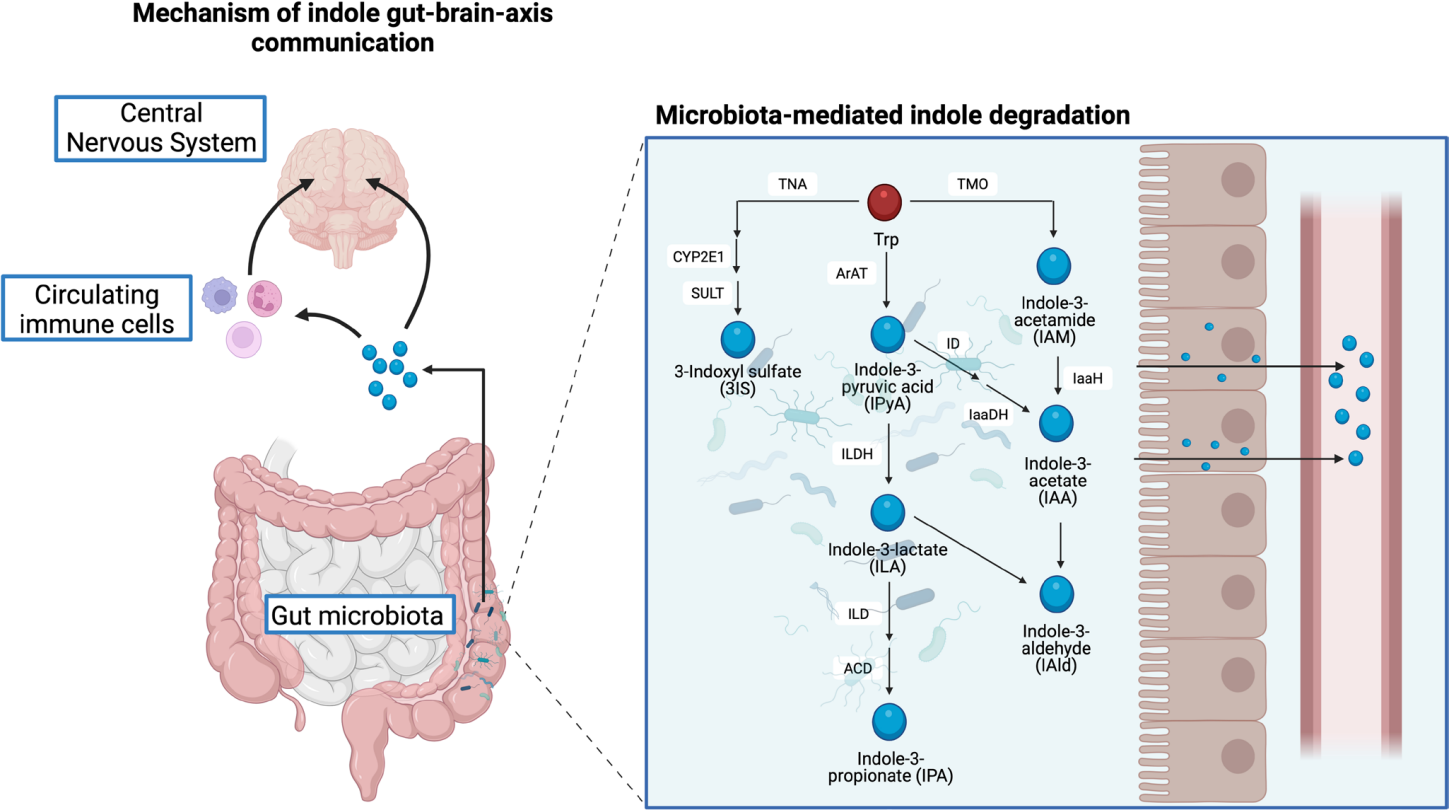
Trp‐derived indole degradation pathways in the gut microbiota and gut‐brain‐axis communication of indoles. Trp, tryptophan; TNA, tryptophanase; TMO, tryptophan monooxygenase; CYP2E1, cytochrome P450 2E1; SULT, sulfotransferase; ArAT, aromatic amino acid aminotransferase; IL4i1, interleukin‐4‐induced‐1; IaaH, indoleacetamide hydrolase; ID, indolepyruvate decarboxylase; IaaDH, indoleacetaldehyde dehydrogenase; ILDH, indolelactate dehydrogenase; ILD, indolelactate dehydratase; ACD, acyl‐CoA dehydrogenase. Created with Biorender.com.

Indoles are tryptophan (Trp)‐derived metabolites that contain a structurally stable heterocyclic aromatic pyrrole ring fused with a benzene ring [1], and are ubiquitously distributed across multiple taxa [2, 3]. In the human gut, indoles are exclusively generated from Trp by specific bacterial genera containing enzymes that mediate each step of indole catabolism [4, 5]. This bacterial‐mediated catabolism results in the generation of indoles following three main routes: generating 3‐indoxyl sulfate (3IS); generating indole‐3‐pyruvic acid (IPyA), indole‐3‐lactate (ILA), and indole‐3‐popionate (IPA); and generating indole‐3‐acetamide (IAM), indole‐3‐acetate (IAA), and indole‐3‐aldehyde (IAld) (Figure 1).

Indoles in the human body are solely produced by indole‐producing bacteria in the gut [6], and thus circulating levels of indoles reflect the bacterial enteric composition and metabolism, and the capability of indoles to translocate into the bloodstream. Both the composition and activity of enteric bacteria and changes in gut permeability are known to be influenced by acute and chronic exercise [7]. Indoles are emerging as key mediators of the gut‐brain axis, as evidenced by their ability to cross the blood–brain barrier and subsequent presence in the rodent brain and human cerebrospinal fluid [8, 9]. Consequently, indole metabolism is beginning to garner attention in neurological diseases such as multiple sclerosis (MS) [4]. MS is a neuroinflammatory autoimmune disease characterized by the activation of adaptive cells and innate immune cells. The immune system is influenced by the metabolic energy demands and nutrition, including metabolites derived from the essential amino acid Trp [10].

In persons with MS (pwMS), there is a marked reduction of the relative abundance of bacterial species that produce ILA as well as reduced serum levels of ILA [11]. Particularly ILA is a bioactive molecule with anti‐inflammatory and remyelinating properties, and it is presently being tested as a remyelinating agent in animal MS models, while IAA is an oxidative indole found to inhibit remyelination in vivo [12]. Therefore, the ILA/IAA index correlated negatively with worse MS clinical scores. Jank and colleagues [12], found that a three‐week oral ILA supplementation decreased spinal cord demyelination, reduced immune cell infiltration and inflammation, and diminished reactive astrogliosis and microgliosis, which ultimately resulted in improved experimental autoimmune encephalomyelitis (EAE) clinical scores [12]. In the same study but in a cuprizone‐induced demyelination model, ILA supplementation increased the percentage of myelinated axons and reduced inflammatory glial activation in the corpus callosum [12]. Recently, ILA in stool was negatively linked with a 2 year change in the Expanded Disability Status Scale (EDSS) score [13]. For this reason, ILA is presently at a preclinical stage and undergoing a patent process as a low‐risk treatment option for pwMS (PCT/US2024/027239).

Regular physical exercise is a strongly recommended preventive strategy for pwMS [14], and influences the gut microbiota. Particularly, moderate‐to‐intense endurance exercise has been shown to alter gut microbiota according to BMI status in young adults [15]. Previous exercise studies on Trp metabolism in pwMS have mainly focused on kynurenine pathway metabolites [16, 17, 18]. None of these efforts have yet focused on indoles and their neuroprotective potential. Thus, research is needed to gain a deeper understanding of how exercise interventions affect circulating levels of indoles and their potential role in the gut‐brain axis, and their anti‐inflammatory and remyelinating properties. Here, we report that a 10 week combined multimodal functional training intervention increased the ILA/IAA index and acutely increased circulating levels of the neuroprotective metabolite ILA.

## 2 Methods

### 2.1 Participants

A total of 45 adults with RRMS completed the two‐armed randomized controlled trial of a 10‐week multimodal functional training with a usual care waitlist control group. In this analysis, we considered participants with (≥ 70% compliance, *n* = 31). Inclusion criteria to participate in the study were as follows: MS diagnosis according to the 2010 revised McDonald criteria [19], RRMS phenotype, EDSS score ≤ 4.0 (mild), adult age (≥ 18 years), and both resting and exercise electrocardiographic clearance (Custo Diagnostic, custo med GmbH, Ottobrunn, Germany), performed by a certified medical doctor. Individuals who presented with an acute episode (relapse) or exacerbation of MS symptoms, or that had cardiovascular or orthopedic diseases, concomitant or a previous history of disease states affecting study endpoints (e.g., cancer), were excluded from participation. Pregnant and breastfeeding women were excluded. A detailed account of the participant characteristics can be found in (Table 1). Furthermore, a control group matched for sex, age, and BMI was included to enable a comparison of baseline values. Heathy‐matched controls were derived from other German exercise studies (DRKS00031445, DRKS00028792, DRKS00029105). Blood samples of the RRMS cohort and the healthy‐matched controls were analyzed in the same lab.

**TABLE 1:** Participant baseline characteristics.

	Healthy matched controls	RRMS total	RRMS control	RRMS intervention
No. of participants	31	31	20	11
Sex F:M	24:7	23:8	14:6	9:2
Age	38.81 (7.78)	38.13 (6.64)	38.30 (7.59)	37.82 (4.77)
BMI	24.61 (3.20)	25.00 (4.81)	25.51 (3.59)	24.06 (6.59)
Fatt mass %	22.47 (3.78)	31.17 (8.15)	32.18 (8.04)	29.34 (8.39)
VO_2peak_	36.34 (6.03)	32.59 (10.12)	32.41 (10.90)	32.96 (9.19)
EDSS score		1.55 (1.14)	1.47 (1.47)	1.68 (1.12)
TSD (years)		6.67 (6.83)	7.21 (6.76)	7.68 (7.26)
Previous relapses (total number)		3.3 (3.01)	3.58 (3.37)	2.82 (2.32)
DMT treated		28 (90.3%)	18 (90.0%)	10 (90.9%)
Anti‐CD20		7 (22.6%)	4 (20.0%)	3 (27.3%)
Platform therapy (interferon beta and glatiramer acetate)		6 (19.4%)	4 (20.0%)	2 (18.2%)
Anti‐α4‐integrin		5 (16.1%)	5 (25.0%)	0 (0.0%)
Fumarates		6 (19.4.%)	2 (10.0%)	4 (36.4%)
S1PR modulators		3 (9.7%)	2 (10.0%)	1 (9.1%)
Teriflunomide		1 (3.2%)	1 (5.0%)	0 (0.0%)
No DMT		3 (9.7%)	2 (10.0%)	1 (9.1%)
Smoker		2 (6.5%)	1 (5.0%)	1 (9.1%)

*Note:* Participant baseline information. Continuous data are given as Mean (SD). Categorical data are given as total numbers (%). Platform therapies include interferon beta and glatiramer acetate treatment.

Abbreviations: BMI, body mass index; DMT, disease‐modifying treatment; EDSS, Expanded Disability Status Scale; RRMS, relapsing–remitting multiple sclerosis; S1PR, Sphingosine‐1‐phosphate receptor; TSD, time since diagnosis; VO_2peak_, peak oxygen uptake.

This secondary analysis is derived from the original study which was approved by the ethics committee of the German Sport University Cologne (ID: 109/2018; October 4, 2018) and registered at the German Clinical Trials Register (DRKS00017091; April 5, 2019) based on the original study protocol [20]. Study conduction was adapted to accommodate the circumstances of the Coronavirus Disease 2019 (Covid‐19) pandemic [20]. Prior to their involvement in the study, all participants were informed and provided written consent in accordance with the Declaration of Helsinki.

### 2.2 Cardiopulmonary Exercise Testing and Randomization

**FIGURE 2:**
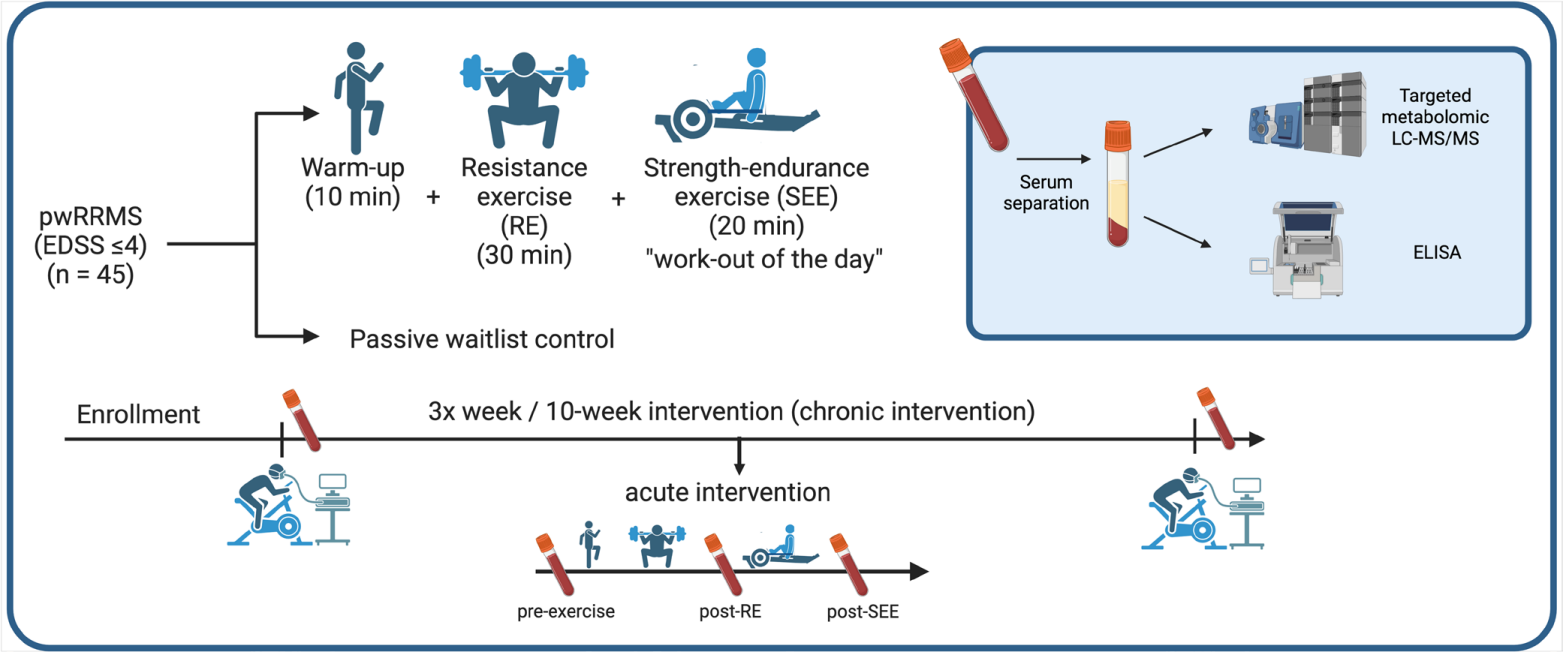
Exercise intervention design, sample collection and analysis. Created with Biorender.com.

Cardiopulmonary exercise testing (CPET) was performed at baseline and at the end of the 10‐week intervention (see Figure 2). The CPET was designed as a progressive ramp protocol and performed on a stationary bicycle (ErgoSelect 5, Ergoline, Bitz, Germany) to determine the HRmax and VO_2peak_ using the yzer 3B (CORTEX Biophysik, Leipzig, Germany). CPET was performed until complete exhaustion (RPE 19–20) or in case of any symptom limitation related or unrelated to MS. Randomization was performed using the Randomization‐in‐Treatment‐Arms software (Evidat, Germany) by a person not involved in the study. The stratification factors were age, EDSS, sex, relative peak power output (PPO), relative leg strength, DMT use (yes/no), and lean body mass. Due to the nature of the trial design, participants were not blinded to group allocation, and post intervention outcomes were performed by sports scientist assessors blinded to group allocation.

### 2.3 Training Program

The training program entailed a community‐based, supervised multimodal functional training workout comprising both strength and aerobic exercises, with a session duration of 60 min, a frequency of 3× per week, and an intervention duration of 10 weeks (Figure 2).

The first phase of the session consisted of a 10 min warm‐up on a ski ergometer or rowing machine, followed by dynamic stretching and core stability exercises. The next phase of the session consisted of a 30 min progressive resistance exercise (RE) phase involving free weight exercises (squat, pull‐up, deadlift, bench press) where intensity was progressively increased based on the one‐repetition maximum (1RM). The final phase consisted of “work‐out of the day”, designed as a 20 min progressive, combined strength and endurance exercise (SEE) phase of interval type (Figure 2). The intensity of each training phase was measured both subjectively and objectively using the rate of perceived exertion 6–20 scale (RPE 6–20) [21], at the end of each training phase and heart rate (HR) monitoring throughout the complete session (Table S1). A detailed description of SEE can be found in Proschinger et al. [22].

### 2.4 Outcome Assessments

All assessments were performed at the German Sport University Cologne between 7:00 am and 4:00 pm in non‐fasted conditions. Participants were asked to refrain from exercise 24 h prior to the assessments.

Venous blood samples were collected at baseline and after 10 weeks to assess chronic effects of the intervention. Samples were collected at resting state before the CPET To assess acute effects in response to a single exercise bout, samples were also taken before, immediately after the REE phase, and immediately after the SEE phase at a community‐based gym (see Figure 2). Blood samples were drawn from the median cubital vein by a qualified technician into serum tubes (8 mL Vacutainer, Becton Dickinson GmbH, Heidelberg, Germany) or BD Vacutainer CPT tubes (8 mL Vacutainer, Becton Dickinson GmbH, Heidelberg, Germany) for serum samples and peripheral blood mononuclear cell (PBMC) isolation, respectively. For the interim measurement, samples were transported directly to the German Sport University Cologne and immediately processed. For the assessments, serum samples were rested for at least 30 min in an upright position and subsequently centrifugated (Megafuge 3.0R., Heraeus, Germany) at 3500 g for an additional 10 min. Thereafter, serum samples were aliquoted and frozen at −80°C until further analysis. For PBMC isolation, density gradient tubes with Ficoll‐Plaque were centrifugated for 20 min at 1700 g. After isolation, the cells were washed twice with phosphate‐buffered saline (PBS) and resuspended in freezing buffer. Viability was assessed using [Trypan Blue/Live‐Dead Stain], and the number of viable cells were used for aliquoting the samples. The samples stored with freezing aid boxes at −80°C. Over the next 24 h, the samples were transferred to a 150°C freezer.

### 2.5 HPLC–MS/MS

Serum concentrations of Trp, indoles and neopterin ‐a marker of immune cell activation‐were determined at baseline, in an acute interim measurement, and after the 10‐week intervention (Figure 2) using high‐performance liquid chromatography coupled with tandem mass spectrometry (LC–MS/MS). Details on LC–MS/MS procedures have been published previously [23]. Metabolites (3IS, ILA, IAA, IAld, and IPA) not initially included in the original validated method were added individually along with their isotope labeled internal standard and the same validation procedures were performed (incl. linearity testing, accuracy, precision, and recovery), followed by cross‐validation of the original assay ensuring quantitation and chromatographic performance were unaffected. Samples were first treated with acetonitrile, sonicated, and centrifuged for protein precipitation. The supernatant was treated with aqueous ammonia and injected into an ACQUITY UPLC system coupled to a Xevo TQ‐XS mass spectrometer. Chromatographic separation was achieved using a Gemini C6‐Phenyl reversed‐phase analytical column with gradient elution (Phenomenex, Aschaffenburg, DEU). Ionization was performed in positive mode, and multiple reaction monitoring (MRM) transitions were used for the quantification of Trp, 3IS, ILA, IAA, IAld, and IPA. The ILA/IAA was then calculated from these values.

### 2.6 Elisa

Serum levels of interleukin‐6 (IL‐6) were quantified using an enzyme‐linked immunosorbent assay (ELISA) kit (R&D systems, USA) following the manufacturer's protocols. Absorbance was measured at 450 nm and 560 nm as reference wavelengths using a microplate reader.

### 2.7 Statistical Analysis

**FIGURE 3:**
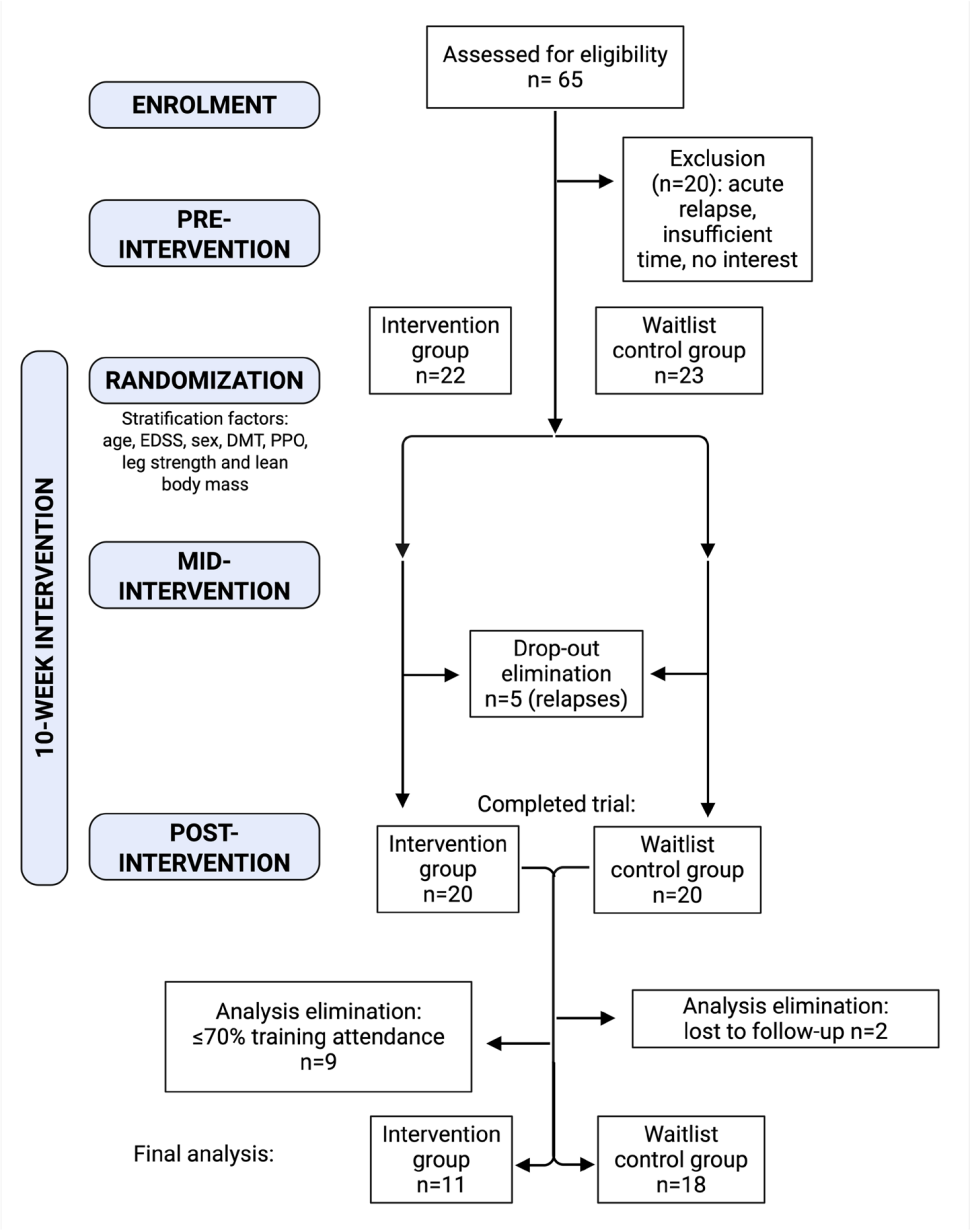
Study design. EDSS, Expanded Disability Status Scale; DMT, disease‐modifying treatment; PPO, peak power output. Created with Biorender.com.

All statistical analyses were conducted using IBM SPSS Statistics (version 29.0.2.0), and a *p*‐value cut‐off of 0.05. All data from participants with a > 70% training attendance and no relapses during the intervention were included in the analysis, as detailed in (Figure 3). Outliers were then defined as values with Z‐scores ≥ Ι3Ι and removed before performing any analysis to reduce the influence of extreme values.

Independent *t*‐tests with two‐sided *p*‐value determination were used to compare baseline BMI, VO_2peak_, anthropometric values, IL‐6, neopterin, Trp, and indole concentrations of RRMS vs. the matched healthy cohort. Cohen's d was calculated to assess effect sizes for the independent *t*‐tests. Then, a linear mixed‐model analysis was used to assess the acute effects of a single interim exercise bout and the chronic effects of the 10‐week intervention. This method enabled the evaluation of group‐by‐time interactions and time effects, with all models accounting for baseline BMI measurements within individuals. Partial eta squared (ηp^2^) was reported to indicate the magnitude of main effects in the group‐by‐time and time mixed‐model analyses. The analyses were conducted using the restricted maximum likelihood (REML) estimation method. Post hoc comparisons for the group‐by‐time interaction as well as pairwise comparisons were Bonferroni‐adjusted for multiple testing.

## 3 Results

Table 1 shows the baseline characteristics of the studied RRMS sample and the matched healthy controls in total and divided into control and intervention groups. The studied RRMS sample comprised adults with an average age of 38.13 (±6.64) years, 74% females, with a mild disease severity (EDSS) score, and an average time since diagnosis of 6.67 (±6.83) years. Predominant disease‐modifying treatment approaches exhibited a preponderance of therapies preferred for mild courses of MS (platform therapy, fumarate, and teriflunomide; *n* = 13) and those preferred for active forms of MS (anti‐CD20, S1PR modulators, and anti‐α 4‐integrin antibody; *n* = 15). Only three participants were not receiving any disease‐modifying therapy, and treatment distribution between groups was generally balanced. Additionally, anthropometric characteristics of healthy matched controls are presented in (Table 1).

The training design and measurement timepoints are depicted in (Figure 2). The participants underwent a high‐intensity multimodal functional training. Each exercise session consisted of a 10 min warm‐up followed by dynamic stretching and core stability exercises. The resistance exercise (RE) phase of the session was performed at an average rating of perceived exertion (RPE) of 15.57 (±1.81) and an average percentage of maximum heart rate (HR_max_) of 68.59% (±13.06%). The consecutive strength‐endurance exercise (SEE) phase was performed at an average of 84.85% HR_max_ (±17.54%) and an average RPE of 17.26 (±1.33). The performance characteristics are provided in Table S1.

### 3.1 Baseline Indole Concentrations Are Different in Persons With RRMS Compared to Healthy Controls

**FIGURE 4:**
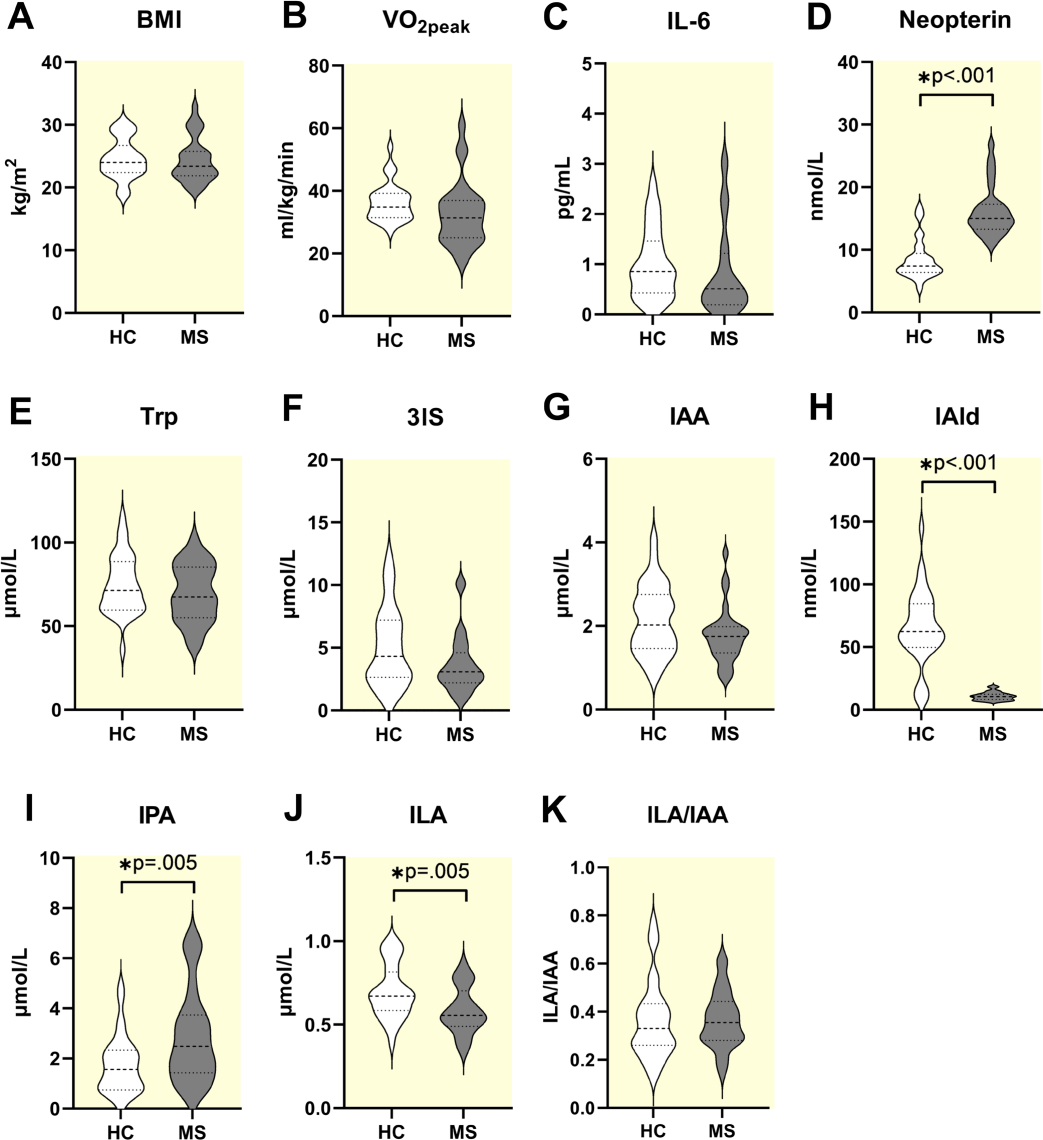
Baseline comparisons of BMI, VO_2peak_, inflammatory markers and Trp‐derived indoles. BMI, body mass index; VO_2peak_, peak oxygen uptake; Trp, tryptophan; 3IS, 3‐indoxyl sulfate; IAA, indole‐3‐acetate; IAld, indole‐3‐aldehyde; IPA, indole‐3‐propionate; ILA, indole‐3‐lactate. * Shows statistical significance for independent t‐test with significance established when *p* ≤ 0.05. Additional statistical results are provided in Table S2.

BMI and VO_2peak_ did not differ between persons with RRMS and healthy controls at baseline (Figure 4A,B). Neopterin levels were higher in persons with RRMS (Figure 4D). Trp levels did not differ between groups (Figure 4E). The comparison of baseline indole levels revealed important initial disparities. IAld (*p* < 0.001) (Figure 4H) and ILA (*p* = 0.005) (Figure 4J) levels were lower in the RRMS group, while IPA levels were higher (p = 0.005) (Figure 4I). The Trp, 3IS, IAA, and the ILA/IAA index did not differ between groups. The complete statistics can be observed in Table S2.

### 3.2 The ILA/IAA Index Increases in Response to 10 Weeks of Multimodal Functional Training

**FIGURE 5:**
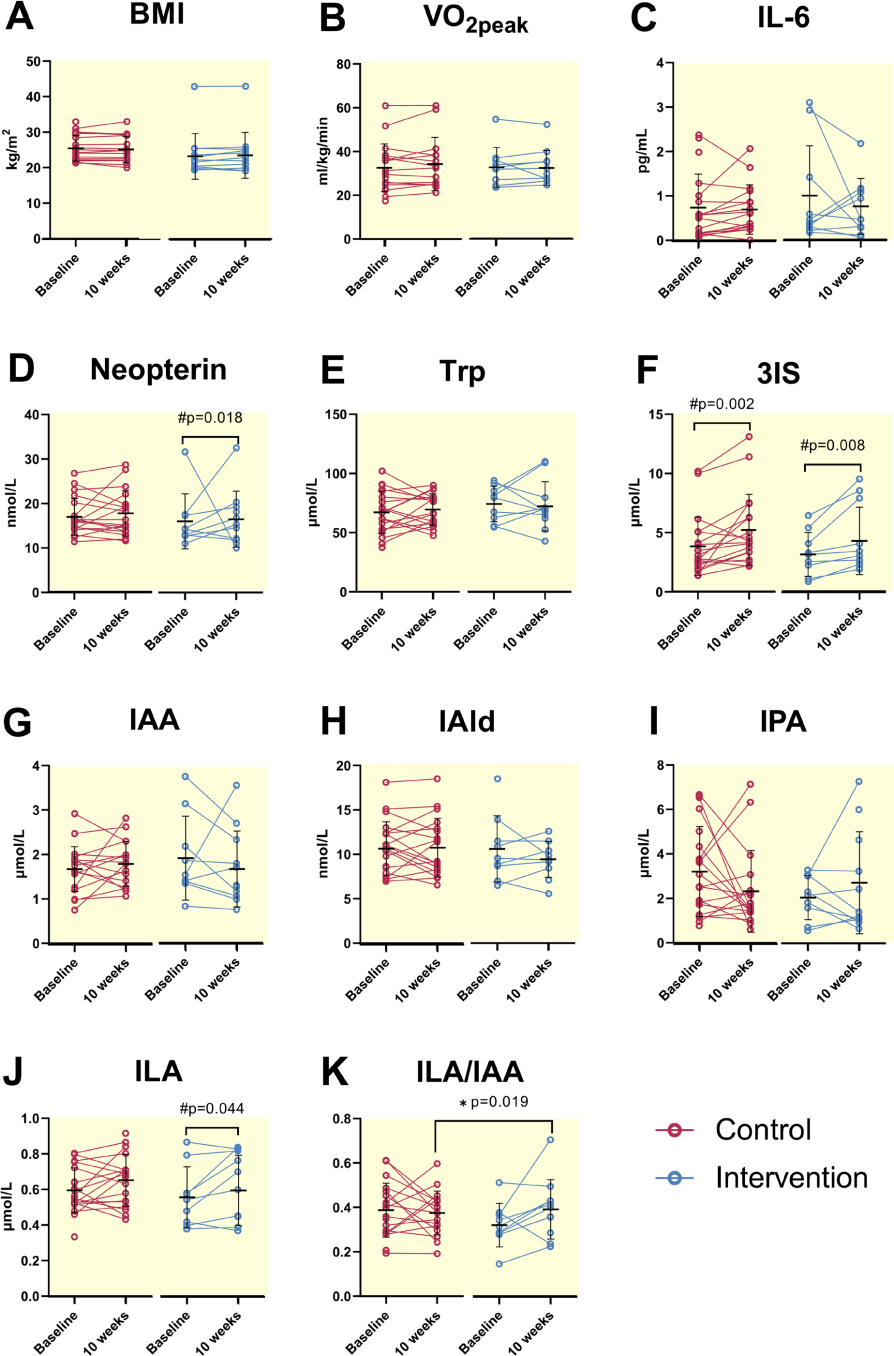
Chronic effects of the 10‐week multimodal functional training on BMI, VO_2peak_, inflammatory markers and Trp‐derived indole levels. BMI, body mass index; IL‐6, interleukin 6; Trp, tryptophan; 3IS, 3‐indoxyl sulfate; IAA, indole‐3‐acetate; IAld, indole‐3‐aldehyde; IPA, indole‐3‐propionate; ILA, indole‐3‐lactate. *Shows statistical significance for Bonferroni‐corrected pairwise comparisons when group‐by‐time interactions were significant, and # indicates Bonferroni‐corrected pairwise comparisons when time effects were significant. Significance was established at *p* < 0.05. Detailed statistical results can be observed in Table S3a,b.

No group‐by‐time interactions or time effects were found in BMI and VO_2peak_ values (Figure 5A,B). Similarly, no group‐by‐time interactions were observed for levels of the inflammatory markers IL‐6 and neopterin or levels of individual indoles (Figure 5C–J). The ILA/IAA index changed over time in response to the intervention (*p* = 0.049) (Figure 5K). A detailed description of group‐by‐time effects and time effects can be found on Table S2. Additionally, time effects were observed for 3IS (*p* < 0.001) and ILA (*p* = 0.026), with increasing levels of both metabolites over time (Table S3b).

### 3.3 A Single Exercise Bout Decreases Trp and Increases ILA Levels

**FIGURE 6:**
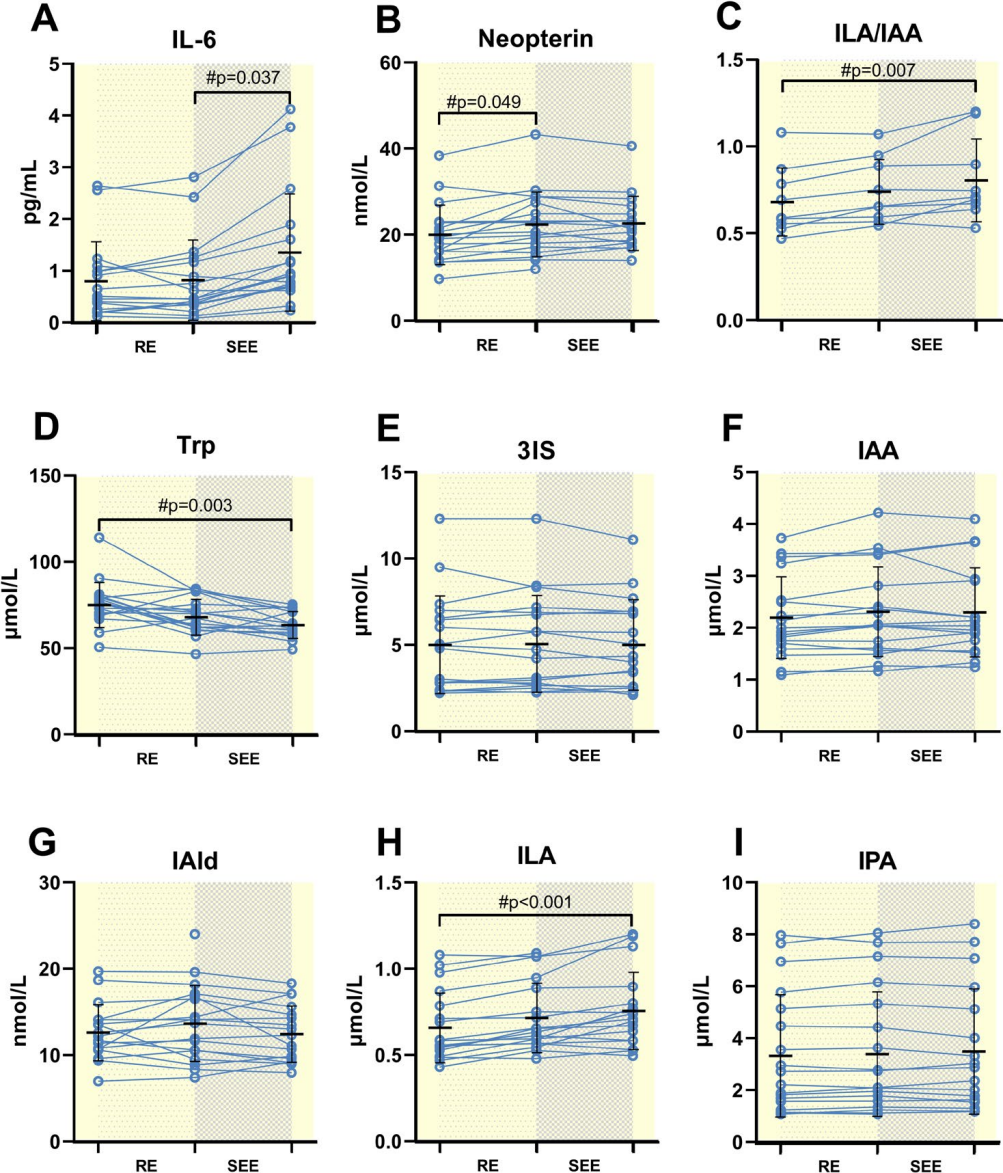
Acute effects of a single exercise bout, including a resistance (RE) and strength endurance (SEE) phase, on inflammatory markers and Trp‐derived indole levels. IL‐6, interleukin 6; Trp, tryptophan; 3IS, 3‐indoxyl sulfate; IAA, indole‐3‐acetate; IAld, indole‐3‐aldehyde; IPA, indole‐3‐propionate; ILA, indole‐3‐lactate. # Indicates Bonferroni‐corrected pairwise comparisons when time interactions were significant. Significance was established at *p* < 0.05. Detailed statistical results can be observed in Table S4.

The acute effects of a single bout of exercise can be observed in (Figure 6). IL‐6 levels did not show any change following the RE phase but increased after the SEE phase (*p* = 0.037) (Figure 6A). Increased neopterin levels were shown already after the training (*p* = 0.049) (Figure 6B).

A statistically significant decrease in Trp levels was observed in response to the exercise bout when comparing before and immediately after the exercise bout (Figure 6D). Among the indoles, only the ILA level was significantly altered by the training session. The ILA serum concentrations gradually increased during the training session, and significant differences were noted between before and immediately after the exercise bout (*p* < 0.001) (Figure 6H). The ILA/IAA index demonstrated a comparable increase, which was significant when comparing the same timepoints (*p* = 0.007) (Figure 6C). A detailed description of the acute effects of a single exercise bout can be found on Table S3.

## 4 Discussion

This study aimed to measure the acute and chronic changes in Trp‐derived indoles after a single bout and 10 weeks of multimodal functional training. To our knowledge, this is the first study to investigate the effect of exercise on indoles in pwMS. We observed that (1) IAld and ILA serum indoles are decreased in pwMS compared to healthy controls, (2) the exercise program increases the ILA/IAA index, reflecting potential beneficial, neuroprotective microbiome changes; and (3) a single bout of exercise acutely increases circulating levels of ILA, an indole with remyelinating properties.

Exercise is known to elicit numerous physiological changes, which have increasingly been recognized as influencing the complex communication within the gut‐brain axis. These responses are initiated by the acute stimulation of the sympathetic nervous system at the onset of exercise, which in turn elicits alterations in the cardiovascular, respiratory and endocrine systems. These alterations are essential for sustaining the body's homeostasis in elevated energy demands [24]. As a result, body temperature is increased and blood flow is redirected to the skeletal muscle and reduced in splanchnic organs such as the gut, transiently increasing the gut permeability [7]. A transient increase in intestinal permeability may therefore facilitate the exchange of metabolites between the gut lumen and the systemic circulation. Conversely, chronic exercise improves indicators of gut barrier integrity, and gut barrier‐promoting bacterial populations [7]. Moreover, chronic exercise has been shown to enhance gut microbial diversity which could potentially modulate local mucosal immunity and therefore increase the production of metabolites derived from bacterial metabolism [25].

It is generally accepted that microbiota diversity is a positive hallmark of gut functionality and stability [26], and that it is typically shaped in response to chronic environmental changes, such as physical activity [27]. Currently, the composition and function of gut microbiota are studied with great interest as microbiota influence (neuro)immune function through various mechanisms [28], and may thus have therapeutic potential. There is a consensus that alpha diversity, which reflects within‐group diversity, is similar in pwMS when compared to healthy individuals [29, 30]. Some studies show MS‐specific differences in community structure as reflected by beta diversity, which reflects the community structure, particularly in relation to differential disease‐modifying treatment use [31, 32]. Nevertheless, a distinct taxonomic abundance seems to be the signature of the MS gut microbiota [29, 30, 31, 32, 33, 34], even if not reflected in the community and diversity analysis [28]. Taxonomic abundance is additionally related to MS progression [28]. Berer et al. [35], found that in an adapted EAE model resembling a relapsing–remitting phenotype (“RR model”) [36], spontaneous EAE occurred more frequently in mice transplanted with microbiota from pwMS compared to microbiota transplanted from their healthy twins.

The differences in taxonomic abundance include a reduction in *Lactobacillus* [33], which together with Bifidobacteria ALDH+ are the main known ILA‐producing bacteria [37, 38]. Therapeutic strategies aimed at increasing ILA production in the gut microbiota by increasing the abundance of these taxa are currently being tested in animal models. Bianchimano et al. [39], found that EAE mice treated with vancomycin increased serum ILA and *L. reuteri* , which are taxa with the capacity to synthesize ILA. They also found increased transcription of mTOR pathway genes in astrocytes [39], implying that the mechanistic link between mediating the neuroimmune benefits of increased ILA production in the gut is the activation of the aryl hydrocarbon receptor (AhR) in the central nervous system (CNS).

Other mechanisms through which ILA modulates immune cells include the phosphorylation of signal transducer and activator of transcription 3 (STAT3), which leads to a reduction in the HK2 metabolic pathway, as observed in vivo in colorectal cancer cells [40]. As indoles act as dual ligands, ILA may also mediate anti‐inflammatory effects, particularly in circulating immune cells, by modulating Nuclear Factor Erythroid 2‐Related Factor 2 (Nrf2) [41]. This has been observed in Nrf2 knockout mice under ischemia/reperfusion injury, and in patients undergoing surgery with a high risk of acute mesenteric ischemia [42]. Both STAT3 and Nrf2 signaling mechanisms are plausible mechanisms, as observed in other pathophysiological contexts. However, their relevance in MS pathogenesis and particularly the exercise‐induced activation of these pathways remains to be proven.

Interestingly, a 10‐week pilot study on aerobic exercise in overweight young adult women found increased group‐by‐time interactions in Bifidobacteria and increased time effects in *Lactobacillus* [43]. Additionally, the Bifidobacteriaceae family increased in previously sedentary and overweight adult women who participated in a 6 week moderate endurance exercise program [44]. ILA levels were not investigated in this study. However, an accompanying increase in ILA production could be expected, derived from an increased abundance of ILA‐producing bacteria. Exercise could therefore increase the enteric production of indoles and/or change gut membrane permeability, thereby increasing the circulating concentration of indoles, thus enhancing their availability to act on key targets in circulating immune cells and the CNS, although clear evidence of ILA crossing the blood‐brain barrier is still lacking. In response to our multimodal functional training intervention, we did not find increased serum levels of ILA. Instead, time effects were identified with ILA increasing in both groups. We additionally found an increased ILA/IAA index in response to the 10 week multimodal program. This index is starting to be recognized along with the clinical relevance of the microbiome in MS symptomatology, as recent evidence in the EAE mouse model demonstrates correlations between a lower ILA/IAA and higher disability level, as indicated by the cumulative EAE clinical score [12]. The ILA/IAA would therefore serve as a valuable metric for assessing the efficacy of exercise in mitigating symptoms in people with MS, with these benefits being attributable to alterations in the gut microbiota.

Keeping in mind the differential effects of a single bout of exercise (acute effect) and training programs (chronic effects) on the immune system [45] and Trp metabolism [46], it makes sense to additionally explore potential acute effects of exercise on gut microbiota as a mediator of some of these benefits. In a sophisticated approach, Tabone et al. [47], assessed the acute effects of a single bout of exercise consisting of a 10 min warm‐up on a treadmill at 60% of HR_max_, followed by a maximum aerobic effort until exhaustion in healthy, adult cross‐country skiers. Stool samples were collected within 4 h after exercise without interim food intake. Interestingly, the authors found an increase in Trp biosynthesis and bacterial taxa with Trp biosynthesis capacity in stool samples after the exercise bout [47]. Therefore, in addition to the Trp that is ingested and reaches the colon, increased local biosynthesis of Trp within the colon would increase the availability for catabolism through the indole pathway, in turn increasing circulatory levels of indoles.

An increase in the inflammatory markers IL‐6 and neopterin was observed following an acute bout of SEE, consistent with previous findings in pwMS [48]. In agreement with the well‐established effects of acute exercise on Trp metabolism [49], a reduction in serum Trp levels and an increase in ILA after a single bout of exercise also occurred. In rodents, ILA serum levels are primarily derived from ILA‐producing bacteria in the gut and are dependent on Trp availability [37]. AhR activation was suggested as the underlying mechanism, underscored by increased gene expression of *AhR*, *CYP1A1*, *CYP1A2*, *CYP1B1*, and *IL22* in both *postmortem* hippocampal tissue of a mouse model of chronic unpredictable mild stress and in vitro in a neuroinflammation model [37]. In the context of exercise, a single bout of endurance exercise has been shown to activate the AhR in circulating immune cells in healthy young adults in an intensity‐dependent manner [50]. Although the mechanism demonstrating the molecule(s) responsible for AhR activation in this context remains to be proven, as various Trp‐derived metabolites are known agonists of the AhR [51].

It is important to note that the physiological and pathophysiological concentrations of indoles in the systemic circulation are not yet well understood, neither in healthy individuals nor in pwMS. Rosas et al. [52], assessed differences in plasma concentrations of indoles in 140 healthy controls and persons with Huntington's disease [52]. In comparison, our data show average higher levels of Trp (74.66 μmol/L), ILA (0.70 μmol/L) and IAA (2.15 μmol/L), and a higher level of IPA (1.75 μmol/L). The serum level of the substrate Trp was much higher in our sample, which challenges direct comparisons of indole concentrations with respect to Trp bioavailability.

Our group has previously discussed the potential effects of ILA and other Trp‐derived metabolites on the activation of the AhR in circulating immune cells [53]. The question remains as to whether exercise‐induced changes in serum levels of indoles are sufficient to activate non‐enteric targets, particularly circulating immune cells capable of crossing the blood–brain barrier, as well as CNS‐resident immune cells. Addressing this issue would facilitate a more comprehensive understanding of the mechanisms through which exercise can alleviate symptoms affecting pwMS. In this regard, high‐intensity exercise is likely to be the most effective exercise modality for increasing Trp‐derived metabolites, as evidenced in healthy populations [50], but might be hard to implement in the presence of physical impairments related to higher disability scores in pwMS.

The limitations regarding data collection have previously been delineated in Proschinger et al. [22]. With regards to the present study's specific focus on gut‐derived metabolites, the primary limitation is the absence of stool samples, which hinders the confirmation of whether bacterial abundance or the enzymatic activity of ILA‐producing taxa is altered by acute and chronic exercise. This information would provide a mechanistic understanding of the source of indoles found in circulation [6], as well as differentiate acute from chronic adaptations to exercise. On the other hand, proving the underlying mechanism of action that mediates the anti‐inflammatory potential of ILA in circulating immune cells would confirm the activation of pathways and provide a fuller picture.

Nevertheless, as enteric metabolism is the only source of circulating indoles, it is plausible that changes in microbiota metabolism and gut membrane permeability have direct therapeutic implications for the clinical outcomes of people with pwMS and, to our knowledge, have not been described before in an exercise context.

The training approach in this study was selected due to well‐established holistic benefits in pwMS [14], and the intensity‐dependent effects of exercise on Trp‐derived metabolites [50]. A progressive functional training approach, comparable in intensity to standard HIIT [54], was applied in a supervised setting to enhance cardiometabolic benefits [55]. While VO_2peak_ did not significantly increase after 10 weeks, RPE assessments indicated exertion levels of 15–16 (“hard” to “very hard”). HR during SEE exceeded 80% of HR_max_ calculated at baseline (Table S1), confirming a high intensity intervention [56], lasting at least 20 min. Given the importance of duration at high intensities, extending the SEE phase should be considered in future multimodal training interventions.

## 5 Conclusion

The neuroprotective ILA/IAA index is positively shifted after a 10 week multimodal functional training intervention in persons with RRMS. Additionally, evidence was found suggesting that ILA, an indole metabolite with remyelinating properties in the CNS, is increased in the circulation after a single bout of exercise in pwMS. Collectively, the acute increase of circulatory ILA with every exercise bout and the chronic shift in ILA/IAA may have compensatory neuroprotective effects.

## Author Contributions

T.W.C.: formal analysis, writing – original draft; F.A.: project administration, investigation; S.B.: project administration, investigation; M.W.: project administration, investigation, S.P.: conceptualization, methodology; A.R.: conceptualization, methodology; M.L.S.: conceptualization, methodology; A.S.: investigation; M.K.: writing – review and editing, D.W.: writing – review and editing, C.W.: methodology; M.R.: methodology; F.R.: methodology; T.M.: methodology; A.M.: investigation, methodology; P.M.U.: investigation, methodology; N.J.: supervision, writing – review and editing; P.Z.: supervision, writing – review and editing.

## Funding

This work was supported by Marga und Walter Boll‐Stiftung, 210‐06.01‐19. German Academic Exchange Service, 91865036.

## Conflicts of Interest

The authors declare no conflicts of interest.

## Supporting information


**Data S1:** Supporting Information.

## Data Availability

The data that support the findings of this study are available on request from the corresponding author. The data are not publicly available due to privacy or ethical restrictions.
