# Supplementary material for: Functional Training Mitigates Reduced Circulating Indole‐3‐Lactate Levels in Persons With Relapsing–Remitting Multiple Sclerosis
Source: Acta Physiol (Oxf). 2026 Jan 28;242(3):e70166. doi: 10.1111/apha.70166 (PMC12852534; doi:10.1111/apha.70166)
Supplement: Supplementary file 1 — Data S1: Supporting Information. [file APHA-242-e70166-s001.docx]

**Supplements**

**Supplement Table 1**. Performance characteristics of the 10-week exercise program

|  | Start (resting) | | Resistance exercise (RE) | | Strength-endurance exercise (SEE) | |
| --- | --- | --- | --- | --- | --- | --- |
|  | Absolute | % of maximal* | Absolute | % of maximal* | Absolute | % of maximal* |
| RPE | 7.95 (±2.46) | 39.75 | 15.57 (±1.81) | 77.85 | 17.26 (±1.33) | 86.3 |
| HR (bpm) | 81.55 (±5.89) | 48.67 | 111.79 (±7.98) | 66.71 | 148.41 (±20.30) | 88.57 |

The mean values (±SD) of each participant were calculated throughout the intervention and then averaged across participants to provide a mean value for the complete intervention. *Considering a maximum of 20 in the RPE Borg Scale (6-20) and the HRmax recorded at the baseline cardiopulmonary exercise test (CPET). HR, heart rate; RPE, rating of perceived exertion; BPM, beats per minute.

**Supplement Table 2.** Baseline comparison of RRMS and matched-healthy controls

|  | **Healthy Control** | **RRMS Total** | **Independent t-test** | | | | **Cohen’s *d*** |
| --- | --- | --- | --- | --- | --- | --- | --- |
|  | mean (±SD) | mean (±SD) | P value [CI] | F | t | df | [95% CI] |
| No. of participants | 31 | 31 |  |  |  |  |  |
| Sex F:M | 24:7 | 23:8 |  |  |  |  |  |
| Age (years) | 38.81 (7.78) | 38.13 (6.64) | .714 [-2.997, 4.351] | 0.72 | 0.369 | 60 | .094 [-.405, .591] |
| BMI (kg/m^2^) | 24.61 (3.20) | 25.00 (4.81) | .806 [-1.514, 1.939] | 0.31 | 0.246 | 59 | .063 [-.439, .565] |
| VO_2peak_ (ml/kg/min) | 36.34 (6.03) | 32.59 (10.12) | .091 [-0.617, 8.037] | 4.79 | 1.717 | 57 | .448 [-.072, .963] |
| IL-6 (pg/mL) | 1.00 (0.69) | 0.84 (0.89) | .473 [-0.287, 0.610] | 1.101 | 0.724 | 50 | .201 [-.346, .747] |
| Neopterin (nmol/L) | 8.486 (3.22) | 16.12 (3.91) | **<.001 [-9.511, -5.752]** | 0.581 | -8.134 | 56 | -2.137 [-2.780, -1.482] |
| Trp (μmol/L) | 74.65 (17.51) | 69.60 (17.22) | .264 [-3.924, 14.041] | 0.01 | 1.127 | 58 | .291 [-.291, .799] |
| 3IS (μmol/L) | 5.02 (3.12) | 3.64 (2.29) | .059 [-0.051, 2.809] | 4.54 | 1.931 | 57 | .503 [-.018, 1.019] |
| IAA (μmol/L) | 2.15 (0.82) | 1.77 (0.66) | .054 [-0.007, 0.765] | 3.62 | 1.964 | 58 | .507 [-.009, 1.020] |
| IAld (nmol/L) | 65.24 (29.69) | 10.72 (3.19) | **<.001 [43.419, 65.623]** | 26.89 | 9.831 | 58 | 2.540 [1.849, 3.218] |
| IPA (μmol/L) | 1.75 (1.42) | 2.89 (1.83) | **.005 [-1.920, -0.352]** | 6.18 | -2.899 | 58 | -.749 [-1.270, -.222] |
| ILA (μmol/L) | 0.70 (0.16) | 0.58 (0.14) | **.005 [0.036, 0.196]** | 0.59 | 2.904 | 57 | .757 [.225, 1.284] |
| ILA/IAA | 0.36 (0.16) | 0.37 (0.12) | .862 [-0.081, 0.068] | 1.70 | -0.175 | 56 | -.046 [-.561, .469] |

Independent t-tests assuming equal variances are shown. Cohen’s d value assessing for effect sizes. Continuous data is shown as mean ± standard derivation (SD). BMI, body mass index; IL-6, interleukin 6; Trp, tryptophan; 3IS, 3-indoxyl sulfate; IAA, indole-3-acetate; IAld, indole-3-aldehyde; IPA, indole-3-propionate; ILA, indole-3-lactate VO_2peak_, peak oxygen uptake.

**Supplement Table 3a.** Group-by-time interaction effects of a 10-week functional exercise training in RRMS.

|  | **Fixed effects** | | |  | **Baseline** | | **Pair-wise comparison** | **10-weeks** | | **Pair-wise comparison** |
| --- | --- | --- | --- | --- | --- | --- | --- | --- | --- | --- |
|  | P value | F | error df | ƞp² | Control mean (±SD) | Intervention mean (±SD) | P value [CI] | Control mean (±SD) | Intervention mean (±SD) | P value [CI] |
| BMI  (kg/m^2^) | .246 | 1.405 | 29.026 | 0.046 | 24.886 (0.129) | 24.903 (0.161) | .932 [-.421, .387] | 24.820 (0.129) | 25.178 (0.161) | .090 [-.773, .058] |
| VO_2peak_  (ml/kg/min) | .601 | .266 | 25.884 | 0.010 | 33.137 (0.513) | 33.141 (0.745) | .996 [-1.825, 1.817] | 33.840 (0.599) | 33.170 (0.745) | .487 [-1.254, 2.595] |
| IL6  (pg/mL) | .258 | 1.340 | 24.521 | 0.052 | 0.776 (0.100) | 0.878 (0.135) | .545 [-.442, .237] | 0.819 (0.107) | 0.595 (0.135) | .205 [-.127, .575] |
| Neopterin  (nmol/L) | .087 | 3.161 | 25.849 | 0.109 | 16.184 (0.667) | 16.089 (1.071) | .941 [-2.475, 2.664] | 16.765 (0.706) | 19.777 (1.071) | .025 [.392, 5.631] |
| Trp  (μmol/L) | .912 | .012 | 26.652 | 0.000 | 69.260 (2.432) | 70.848 (3.643) | .720 [-10.443, 7.268] | 71.339 (2.561) | 72.203 (3.643) | .848 [-9.864, 8.136] |
| 3IS  (μmol/L) | .681 | .172 | 26.894 | 0.006 | 3.651 (0.269) | 3.685 (0.403) | .944 [-1.011, .942] | 4.959 (0.284) | 5.275 (0.403) | .526 [-1.311, .679] |
| IAA  (μmol/L) | .330 | .984 | 27.085 | 0.035 | 1.748 (0.102) | 1.818 (0.152) | .706 [-.439, .299] | 1.871 (0.110) | 1.665 (0.152) | .279 [-.172, .585] |
| IAld  (nmol/L) | .941 | .006 | 27.434 | 0.000 | 10.717 (0.315) | 10.626 (0.470) | .873 [-1.048, 1.230] | 10.553 (0.333) | 10.525 (0.504) | .964 [-1.194, 1.250] |
| IPA  (μmol/L) | .320 | 1.027 | 26.143 | 0.038 | 3.009 (0.325) | 2.494 (0.487) | .412 [-.695, 1.669] | 2.195 (0.347) | 2.582 (0.487) | .502 [-1.605, .796] |
| ILA  (μmol/L) | .286 | 1.189 | 25.901 | 0.044 | 0.587 (0.018) | 0.587 (0.027) | .995 [-.065, .066] | 0.617 (0.019) | 0.668 (0.027) | .136 [-.118, .016] |
| ILA/IAA | **.049** | 4.276 | 25.864 | 0.142 | 0.376 (0.020) | 0.357 (0.029) | .569 [-.051,.091] | 0.353 (0.022) | 0.442 (0.029) | **.019** [.015,.163] |

Fixed effects from Linear Mixed-Effects Model Analysis showing group-by-time interactions. Pair-wise comparisons of group-by-time interactions were Bonferroni-corrected. CI shows 95% Confidence Interval for Difference (Lower Bound, Upper Bound). Significance was established when p<.05 and partial eta squared (ηp²) was reported to indicate the magnitude of main effects. BMI, body mass index; IL-6, interleukin 6; Trp, tryptophan; 3IS, 3-indoxyl sulfate; IAA, indole-3-acetate; IAld, indole-3-aldehyde; IPA, indole-3-propionate; ILA, indole-3-lactate; VO2peak, peak oxygen uptake; SD, standard derivation; df, denominator df.

**Supplement Table 3b.** Time effects of a 10-week functional exercise training in RRMS.

|  | **Fixed effects** | | |  | **Control** | | **Pair-wise comparison** | **Intervention** | | **Pair-wise comparison** |
| --- | --- | --- | --- | --- | --- | --- | --- | --- | --- | --- |
|  | P value | F | error df | ƞp² | Baseline mean (±SD) | 10-weeks mean (±SD) | P value [CI] | Baseline mean (±SD) | 10-weeks mean (±SD) | P value [CI] |
| BMI  (kg/m^2^) | .472 | .530 | 29.026 | 0.018 | 24.886 (0.129) | 24.820 (0.129) | .711 [-.293, .424] | 24.903 (0.161) | 25.178 (0.161) | .236 [-.739, .190] |
| VO_2peak_  (ml/kg/min) | .580 | .314 | 25.884 | 0.012 | 33.137 (0.513) | 33.840 (0.599) | .366 [-2.333, .888] | 33.141 (0.745) | 33.170 (0.745) | .978 [-2.187, 2.129] |
| IL6  (pg/mL) | .404 | .722 | 24.521 | 0.029 | 0.776 (0.100) | 0.819 (0.107) | .802 [-.396, .309] | 0.878 (0.135) | 0.595 (0.135) | .219 [-.180, .746] |
| Neopterin  (nmol/L) | **.022** | 5.968 | 25.849 | 0.187 | 16.184 (0.667) | 16.765 (0.706) | .547 [-2.536, 1.374] | 16.089 (1.071) | 19.777 (1.071) | **.018** [-6.701, -.674] |
| Trp  (μmol/L) | .600 | .281 | 26.652 | 0.010 | 69.260 (2.432) | 71.339 (2.561) | .576 [-9.601, 5.442] | 70.848 (3.643) | 72.203 (3.643) | .801 [-12.322, 9.611] |
| 3IS  (μmol/L) | **<.001** | 18.271 | 26.894 | 0.404 | 3.651 (0.269) | 4.959 (0.284) | **.002** [-2.097, -.519] | 3.685 (0.403) | 5.275 (0.403) | **.008** [-2.736, -.444] |
| IAA  (μmol/L) | .914 | .012 | 27.085 | 0.000 | 1.748 (0.102) | 1.871 (0.110) | .446 [-.448, .202] | 1.818 (0.152) | 1.665 (0.152) | .508 [-.316, .622] |
| IAld  (nmol/L) | .758 | .097 | 27.458 | 0.003 | 10.717 (0.315) | 10.553 (0.333) | .731 [-.805, 1.134] | 10.626 (0.470) | 10.525 (0.504) | .888 [-1.353, 1.554] |
| IPA  (μmol/L) | .424 | .659 | 26.143 | 0.025 | 3.009 (0.325) | 2.195 (0.347) | .121 [-.227, 1.832] | 2.494 (0.487) | 2.582 (0.487) | .903 [-1.574, 1.397] |
| ILA  (μmol/L) | **.026** | 5.606 | 25.901 | 0.178 | 0.587 (0.018) | 0.617 (0.019) | .279 [-.085, .026] | 0.587 (0.027) | 0.668 (0.027) | **.044** [-.159, -.002] |
| ILA/IAA | .234 | 1.482 | 25.864 | 0.054 | 0.376 (0.020) | 0.353 (0.022) | .471 [-.041, .085] | 0.357 (0.029) | 0.442 (0.029) | .054 [-.175, .002] |

Fixed effects from Linear Mixed-Effects Model Analysis showing group-by-time interactions. Pair-wise comparisons of group-by-time interactions were Bonferroni-corrected. CI shows 95% Confidence Interval for Difference (Lower Bound, Upper Bound). Significance was established when p<.05 and partial eta squared (ηp²) was reported to indicate the magnitude of main effects. BMI, body mass index; IL-6, interleukin 6; Trp, tryptophan; 3IS, 3-indoxyl sulfate; IAA, indole-3-acetate; IAld, indole-3-aldehyde; IPA, indole-3-propionate; ILA, indole-3-lactate; VO2peak, peak oxygen uptake; SD, standard derivation; df, denominator df.

**Supplement Table 4.** Acute effects of a single bout of functional training.

|  | **Fixed effects** | | | | **Intervention** | | | **Pair-wise comparison**  P value [CI] | | |
| --- | --- | --- | --- | --- | --- | --- | --- | --- | --- | --- |
|  | P value | F | df | ƞp² | Baseline mean (±SD) | Post-RE (±SD) | Post-SEE (±SD) | Baseline vs. post-RE | Baseline vs. post-SEE | Post-RE vs. post-SEE |
| IL6  (pg/mL) | **.021** | 4.953 | 16.000 | 0.236 | 0.589 (0.120) | 0.566 (0.120) | 0.905 (0.129) | 1.000 [-.299, .345] | .055 [-.639, .006] | **.037 [-.661, -.017]** |
| Neopterin  (nmol/L) | **.036** | 4.109 | 16.000 | 0.204 | 16.967 (1.234) | 19.500 (1.234) | 19.056 (1.234) | **.049 [-5.056, -.011]** | .125 [-.4.612, .434] | 1.000 [-2.078, 2.967] |
| ILA/IAA | **.007** | 6.785 | 16.000 | 0.298 | 0.402 (0.049) | 0.419 (0.049) | 0.456 (0.049) | .831 [-.056, .023] | **.007 [-.093, -.014]** | .075 [-.076, .003] |
| Trp  (μmol/L) | **.004** | 8.086 | 16.000 | 0.336 | 77.889 (3.249) | 68.256 (3.249) | 63.411 (3.249) | .055 [-.164, 19.430] | **.003 [4.681, 24.275]** | .614 [-4.952, 14.641] |
| 3IS  (μmol/L) | .623 | .487 | 16.000 | 0.029 | 4.391 (0.660) | 4.501 (0.660) | 4.323 (0.660) | 1.000 [-.596, .376] | 1.000 [-.418, .554] | 1.000 [-.308, .664] |
| IAA  (μmol/L) | .095 | 2.735 | 16.000 | 0.146 | 1.806 (0.240) | 1.878 (0.240) | 1.908 (0.240) | .383 [-.192, .048] | .111 [-.222, .018] | 1.000 [-.150, .090] |
| IAld  (nmol/L) | .204 | 1.756 | 16.000 | 0.099 | 12.177 (1.204) | 13.258 (1.204) | 12.337 (1.204) | .305 [-2.746, .583] | 1.000 [-1.824, 1.504] | .475[-.743, 2.586] |
| IPA  (μmol/L) | -673 | .405 | 16.000 | 0.025 | 3.107 (0.624) | 3.180 (0.624) | 3.162 (0.624) | 1.000 [-.300, .154] | 1.000 [-.283, .172] | 1.000 [-.245, .209] |
| ILA  (μmol/L) | **<.001** | 10.380 | 16.000 | 0.393 | 0.684 (0.072) | 0.741 (0.072) | 0.809 (0.072) | .153 [-.131, .016] | **<.001 [-.198, -.052]** | .079 [-141, .006] |

Fixed effects from Linear Mixed-Effects Model Analysis showing time effects. Pair-wise comparisons of time effects were Bonferroni-corrected. CI shows 95% Confidence Interval for Difference (Lower Bound, Upper Bound). Significance was established when p<.05 and partial eta squared (ηp²) was reported to indicate the magnitude of main effects. BMI, body mass index; IL-6, interleukin 6; Trp, tryptophan; 3IS, 3-indoxyl sulfate; IAA, indole-3-acetate; IAld, indole-3-aldehyde; IPA, indole-3-propionate; ILA, indole-3-lactate; VO2peak, peak oxygen uptake; SD, standard derivation; df, denominator df.
